# Synergistic effect of a drug loaded electrospun patch and systemic chemotherapy in pancreatic cancer xenograft

**DOI:** 10.1038/s41598-017-12670-3

**Published:** 2017-09-28

**Authors:** Eunsung Jun, Song Cheol Kim, Chan Mi Lee, Juyun Oh, Song Lee, In Kyong Shim

**Affiliations:** 1 Division of Hepato-Biliary and Pancreatic Surgery, Department of Surgery, University of Ulsan College of Medicine & Asan Medical Center, 388-1 Pungnap-2 Dong, Songpa-gu Seoul, South Korea; 2 Department of Biomedical Sciences, University of Ulsan College of Medicine, 388-1 Pungnap-2 Dong, Songpa-gu Seoul, South Korea; 3 Asan Institute for Life Science, University of Ulsan College of Medicine and Asan Medical Center, 388-1 Pungnap-2 Dong, Songpa-gu Seoul, South Korea

## Abstract

Pancreatic cancer has a high rate of local recurrence and poor prognosis even with adjuvant chemotherapy after curative resection. The aim of this study was to investigate if local drug delivery combined with low dose systemic chemotherapy can increase the therapeutic effect of chemotherapy while reducing systemic toxicities. Poly-L-lactic acid-based 5-FU releasing patch was fabricated by electrospinning, and its tumour killing effects were first confirmed *in vitro*. The 5-FU patch directly adhered to the tumour in subcutaneous and orthotopic murine models, and induced a significant decrease in tumour size. Systemic gemcitabine treatment group, 5-FU drug releasing patch group, and systemic gemcitabine plus 5-FU patch group were compared by tumour size measurement, non-invasive bio-imaging, and histology in subcutaneous models. Combination of local drug patch and systemic chemotherapy led to increased tumour suppression effects that lasted longer, as well as increased survival rate. Histology revealed higher degree of apoptosis in the combined group. Systemic toxicity was recovered within 7 days after the treatment in all mice. Conclusively, local drug delivery using biocompatible polymer patch significantly inhibited tumour growth, and combination with systemic chemotherapy was more effective than single systemic chemotherapy.

## Introduction

Despite recent advances in cancer research, pancreatic cancer has the poorest prognosis of all solid malignant tumours. This is because sensitive tools for early diagnosis of pancreatic cancer have not been developed, and no effective treatment for advanced pancreatic cancer has been identified to date^1,2^. Despite early diagnosis and curative resection in some patients, pancreatic cancer is associated with a high recurrence rate^3,4^. Inadequate surgical clearance, the microenvironment surrounding pancreatic cancer, and genetic alterations in the tumour itself are factors associated with recurrence^5–7^. Local recurrence after surgery, which occurs frequently and is a unique oncologic feature of pancreatic cancer, determines the survival of patients undergoing surgery. A better understanding of the clinical features of patients and the characteristics of tumours is important to improve the survival of patients with pancreatic cancer, and various pancreatic cancer studies are underway based on these^8,9^.

Systemic chemotherapy based on pathology results is recommended in most patients to prevent tumour recurrence after surgical resection. However, in many cases, the onset of systemic anticancer drugs is delayed or treatment cannot be completed because of postsurgical complications or poor patient conditions^10–12^. Local treatment such as stereotactic body radiation therapy (SBRT) is an alternative to systemic adjuvant therapy, and reports show that the progression of pancreatic cancer is inhibited and the survival rate of patients improves^13–15^. In addition, these treatments improve patient outcomes when combined with systemic chemotherapy^16,17^. Nevertheless, the effect of combination therapy is limited, which prompted our team of experienced surgeons to conduct extensive research in this area^18,19^. We hypothesised that drug delivery through topical patches would be effective immediately after surgery, and our team is developing new therapies based on this hypothesis.

Topical drug delivery through a drug patch could be useful because it may reduce dose frequency and allow intensive delivery to desired sites while avoiding primary metabolism in the liver^20,21^. Drug patches are currently used for the administration of anti-inflammatory, analgesic, or hormonal drugs through the skin^21–24^. Studies are underway to enhance the anticancer efficacy of antibodies or chemicals using various agents such as gels, microneedles, and collagen patches^25–28^. In the case of pancreatic cancer with frequent local recurrence, local patches may be an effective option, although their treatment efficacy remains under investigation.

The aim of the present study was to investigate the therapeutic efficacy of drug patches for the treatment of pancreatic cancer. The drug patches in this study were constructed using an electrospinning method that uses a liquid electrostatic force to generate fine polymeric fibers^29,30^. The therapeutic effect of the patches was verified in *in vitro* and *in vivo* models using pancreatic cancer cell lines, and the results were confirmed by non-invasive bioimaging techniques.

## Result

### Establishment and characterization of drug patch by electrospinning

**Figure 1:**
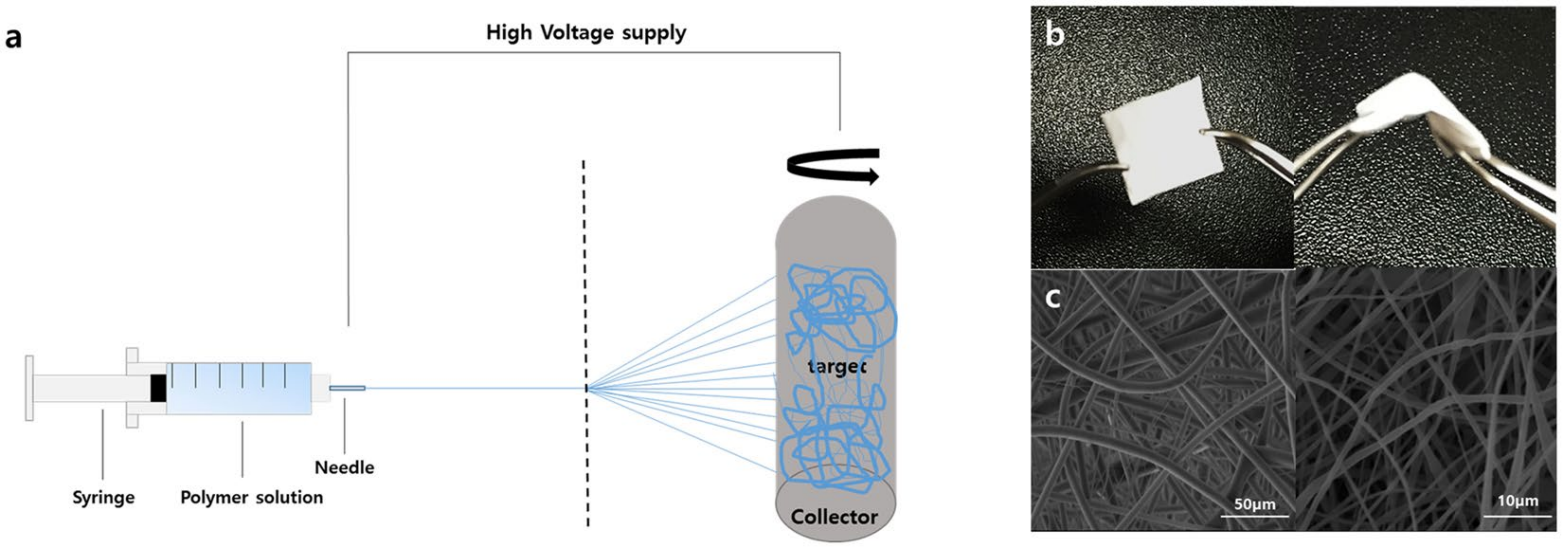
Establishment and morphologic characterisation of a drug patch developed by electrospinning. (**a**) Schematic diagram of the production of the drug patch by electrospinning. A drug patch was prepared using a solution of 20% 5-FU in poly-L-lactic acid. (**b**) The white patch can be adjusted to the desired size and is flexible. (**c**) Scanning electron microscope imaging of the Sham patch (Rt) and 5-FU patch (Lt).

A polymer solution containing 20% fluorouracil (5-FU) in poly-L-lactic acid (PLLA) was injected into a syringe and irradiated with high voltage energy. The irradiated drug-polymer mixture was adsorbed onto the target portion of the collector, and a thin film-like drug-mixed polymer substance was produced through repetition of this process (Fig. 1a). The resulting drug patch had a thin, broad, white surface and a flexible nature, showing resistance to tearing when bent with forceps. The flexibility of the drug patch will allow customisation of its design according to the purpose (Fig. 1b). There were no obvious differences between patches with or without 5-FU. Visualization of the microstructure of the patch using scanning electron microscopy (SEM) confirmed that there were no significant changes in the structure of nanofibers associated with drug absorption (Fig. 1c).

### Therapeutic effect of the 5-FU patch *in vitro*

**Figure 2:**
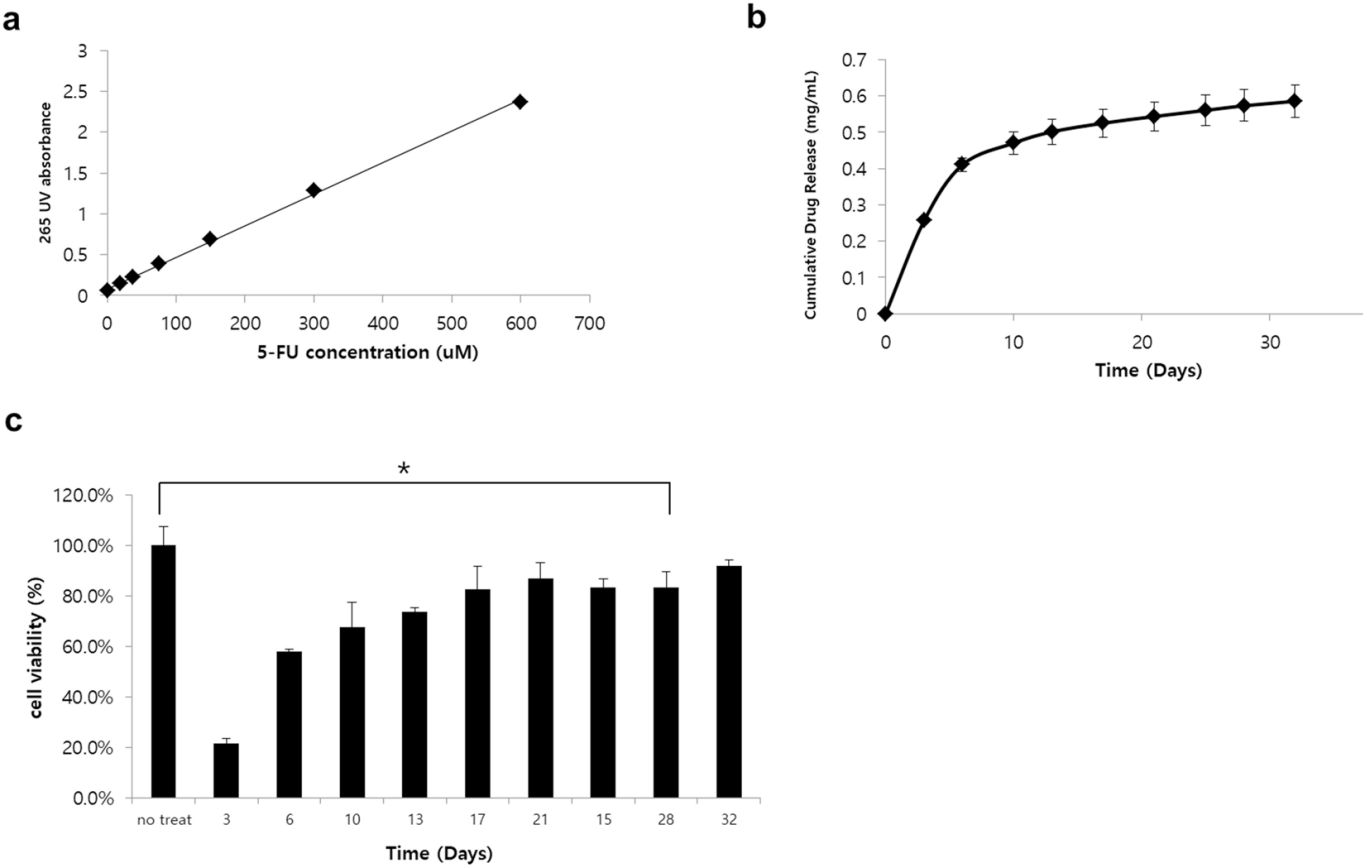
Functional assay of the 5-FU patch *in vitro*. (**a**) The 265 nm UV absorbance of serially diluted 5-FU showed a linear correlation (R^2^ = 0.9988). (**b**) The correlation between UV absorbance and drug concentration was assessed by collecting the media supernatants of the 5-FU patch at different time points. Most of the drug was released within 10 days, and drug release was continuous for up to 30 days. (**c**) The viability of BxPC3-luc cells was investigated using the media supernatants of the drug patches at the different time points. The cytotoxic effect of the released drug was observed predominantly in the early period, and the therapeutic effect was maintained for up to 28 days (*p < 0.05).

To evaluate the drug release profile of the 5-FU patch, a standard curve was generated by measuring the UV absorbance of different 5-FU concentrations at 265 nm. A linear pattern was obtained with a correlation coefficient of 0.9988 (Fig. 2a). The pattern of drug release into the media according to time was then calculated. The results showed that the drug was continuously released over a period of 30 days; however, approximately 80% of the drug absorbed into the patches was released rapidly in the first 10 days (Fig. 2b). To confirm the therapeutic effect of the 5-FU patch, the effect of the medium supernatant of the drug patch on the viability of the BxPC3-luc cell line, which is relatively resistant to 5-FU, was assessed (Sup. Fig. 1, IC 50: 1.15 μM). The results showed that the released drug had a cell killing effect up to 28 days (p < 0.005), and the effect was correlated with the release pattern of the drug shown in Fig. 2b (Fig. 2c). Experiments with a sham patch showed that the patch itself had no cytotoxic effects (Sup. Fig. 2).

### Therapeutic effect of the 5-FU patch in a subcutaneous tumour model

**Figure 3:**
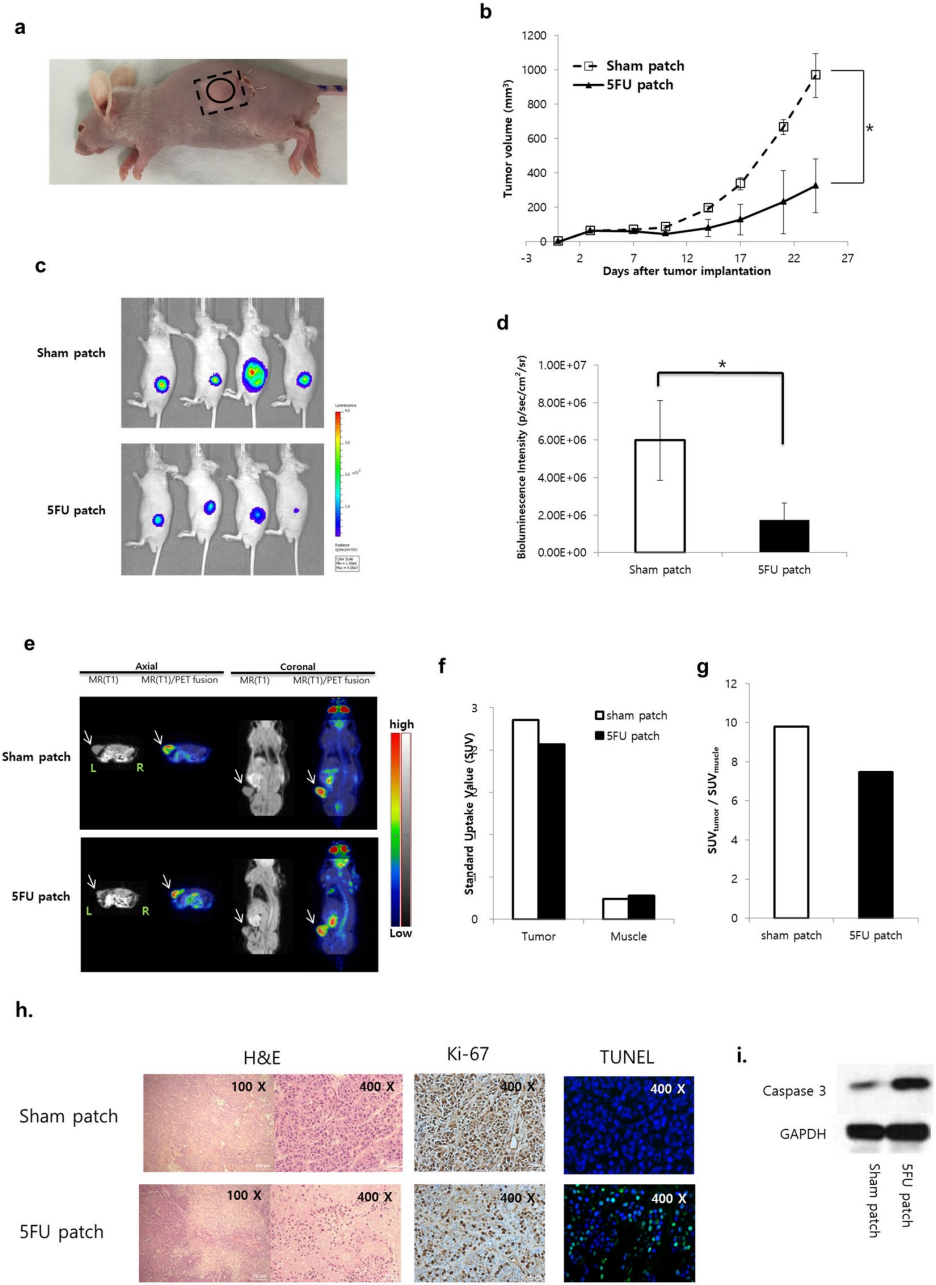
Therapeutic effect of the 5-FU patch in a subcutaneous tumour model. (**a**) The patch was inserted into the mouse flank through a skin incision, and BxPC3-luc cells were inoculated after skin suturing. (**b**) After patch implantation, tumour size was monitored for 25 days. Tumour growth was significantly inhibited in the 5-FU patch group (*p < 0.05). (**c**) Bioluminescence signals were detected in the two groups on day 21 using the IVIS. (**d**) Comparison of the bioluminescence signals between groups demonstrated a positive therapeutic effect in the 5-FU patch group (n = 4, *p < 0.05). (**e**) PET/MRI was performed in the first mouse in each group, and the tumour location (white arrow) and metabolic activity were observed. (**f**) Standard uptake values (SUVs) for mouse tumours and muscle layers around the tumour were quantified. (**g**) Comparison of the ratio of SUV between tumour and muscle indicated that metabolic activity was decreased in 5-FU patch tumours. (**h**) Images of hematoxylin and eosin (H&E)-stained cancer tissue sections from sham patch and 5-FU patch group, along with Ki-67 and TUNEL staining (Tunel;green, DAPI;blue) for each condition. (**i**) Protein expressions of caspase 3 and GAPDH were compared using western blotting.

To confirm the *in vivo* effects of the 5-FU patch, a subcutaneous tumour model was established using BxPC3-luc cells. To insert the patch in the mouse, a square patch measuring 8 × 8 mm (20 mg) was generated. Patches were inserted subcutaneously into the flanks of mice, and BxPC3-luc cells were injected into the upper part of the patch. To prevent leakage of tumour cells from the skin incision site, BxPC3-luc cells were mixed with matrigel and the cells were implanted at a sufficient distance from the incision site (Fig. 3a). Tumour growth was observed over a period of 3 weeks, and the mice treated with the drug patch showed inhibition of tumour growth (Fig. 3b, p < 0.05). The therapeutic effect and anticancer activity of local drug delivery were determined using an *in vivo* imaging system (IVIS) and positron emission tomography/magnetic resonance imaging (PET/MRI). The tumour cells used in this experiment expressed a bioluminescent signal, and the correlation between cell numbers and bioluminescent signals was confirmed before the experiment (R^2^ = 0.9869, Sup. Fig. 3a,b). Tumours in two groups were observed at 21 days after patch implantation in the subcutaneous tumour model. Visualisation of the bioluminescent signal confirmed the therapeutic effect of the 5-FU patch, and the quantitative analysis showed significant differences between the two groups. (p < 0.05, Fig. 3c,d) PET/MRI was used to confirm the effect of the drug on tumour metabolism, and representative images were obtained for the first mouse of each group. MR images showed that tumours were located in the flank, and fusion with the PET image confirmed the metabolic activity of the tumours and surrounding muscles. The glucose metabolic activity of the tumour with the 5-FU patch was 23.8% lower than that of the tumour with the sham patch, indicating decreased proliferative activity (Fig. 3e,f). Tumour size was assessed after mice were sacrificed, which confirmed that 5-FU patch tumours were relatively smaller than sham patch tumours (Sup. Fig. 4). H & E, Ki-67 staining and TUNEL assay using tumour tissues from each group showed decreased cell proliferation and increased cell death in the 5-FU patch group (Fig. 3h). Western blot for caspase 3 also confirmed increase in apoptosis of cancer cells in the 5-FU patch group (Fig. 3i).

### Therapeutic effect of the 5-FU patch in an orthotopic tumour model

**Figure 4:**
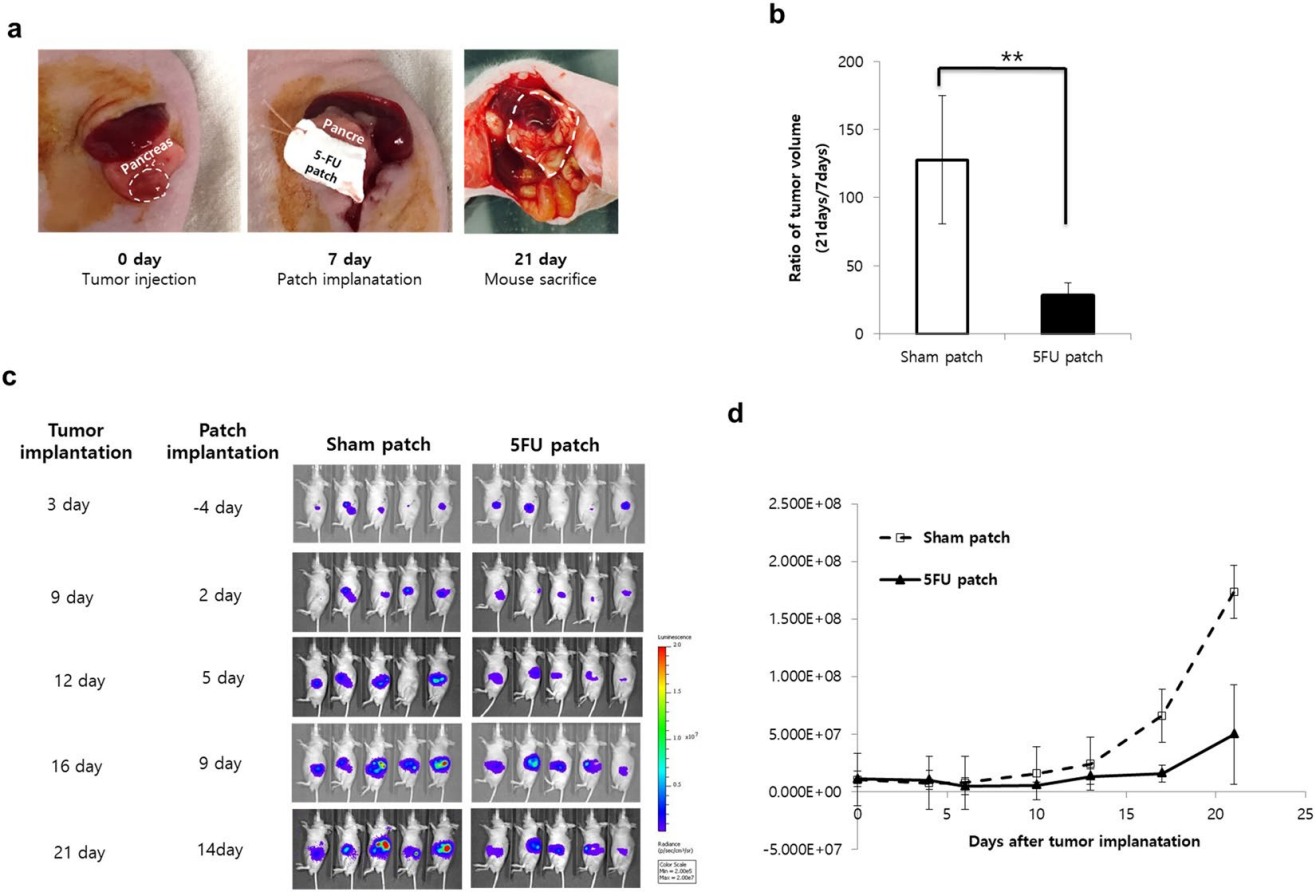
Therapeutic effect of the 5-FU patch in an orthotopic tumour model. (**a**) Tumour cells were inoculated into the pancreas via a small incision around the spleen, and the patch was transplanted 7 days later. On day 21, mice were sacrificed, and the size of the tumours was determined. (**b**) Tumour size was measured in the two groups to confirm the tumour suppressor effect of the 5-FU patch. (**p < 0.01) (**c**) Tumour growth was monitored with the IVIS through day 21 after tumour transplantation. (**d**) Quantification of individual bioluminescence signals confirmed the therapeutic effect in the 5-FU group.

To determine the adhesion of the patch material to internal organs and the efficacy of the drug patch on curved surfaces, a treatment plan was established in an orthotopic pancreatic cancer model. Tumour cells were injected into the pancreatic tail region of mice, and the drug patch was attached after 4 days. PLLA patches adhered easily to the tumour because of their flexibility and stickiness. Patches were marked using sutures to facilitate identification (Fig. 4a). After 17 days, the mice were sacrificed and the tumours were identified. The patch was well attached to the area under the tumour, and mice treated with the 5-FU patch showed dramatic tumour suppression (p < 0.01, Fig. 4b). Because tumour size could not be measured accurately in the orthotopic model, tumours were assessed with the IVIS. Analysis of the bioluminescent signal of tumours in each group showed that the effect of the 5-FU patch was continuous from the beginning of tumour transplantation (Fig. 4c,d).

### Synergistic effect of the 5-FU patch and systemic chemotherapy

**Figure 5:**
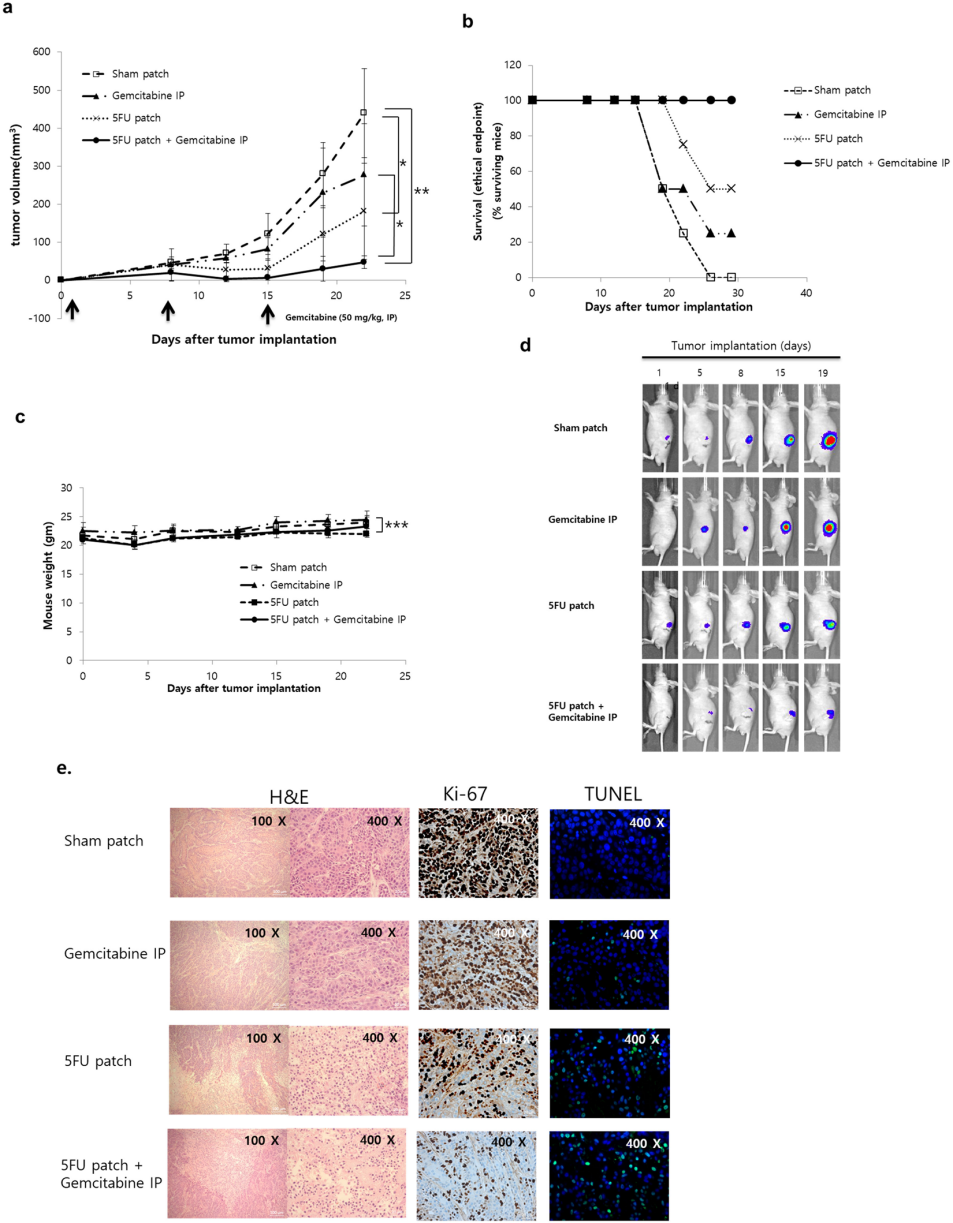
Combination therapy with the 5-FU patch and systemic chemotherapy. (**a**) Subcutaneous models were constructed using BXPC3-luc cells. Mice were subjected to combination treatment with the 5-FU patch and gemcitabine (50 mg/kg, IP injection, once per week). The effect of combination therapy was superior to that of single agent treatment (n = 4, *p < 0.05, **p < 0.01). (**b**) Survival rate of mice in various treatment groups. A tumour volume of 300 mm^3^ was defined as the ethical end point and the survival was presented. (n = 4) (**c**). No differences in body weight were observed between the four groups (***p > 0.05). (**d**) Tumour growth was monitored with the IVIS after tumour transplantation. The bioluminescence signal was lowest in the combination therapy group. (**e**) Images of hematoxylin and eosin (H&E)-stained cancer tissue sections from various treatment groups, along with Ki-67 and TUNEL staining (Tunel;green, DAPI;blue) for each condition.

Finally, we investigated whether the 5-FU patch had a synergistic effect with systemic chemotherapy. The therapeutic effect was compared by adding a systemic injection of gemcitabine, an anticancer agent used for the treatment of pancreatic cancer. As shown in Fig. 5a, the combination of gemcitabine (IP injection, 50 mg/kg, once per week) and the 5-FU patch resulted in an enhanced treatment effect that lasted longer. We also confirmed that survival could be improved by a combination of local 5-FU patch treatment and systemic gemcitabine therapy (Fig. 5b). During the drug administration period, there were no significant differences in body weight between the groups (Fig. 5c). IVIS imaging also showed that the group with combination therapy had the best therapeutic effect (Fig. 5d). H & E staining and TUNEL assay showed that cell apoptosis was significantly increased in 5-FU patch and 5-FU patch + Gemcatabine IP group. On the other hand, cell proliferation was relatively decreased due to the therapeutic effect of the drug (Fig. 5e). Western blot on caspase 3 also confirmed the effects of the drug in the treatment groups (Sup. Fig. 5).

### Evaluation of acute toxicity by hematological and histological analysis

**Figure 6:**
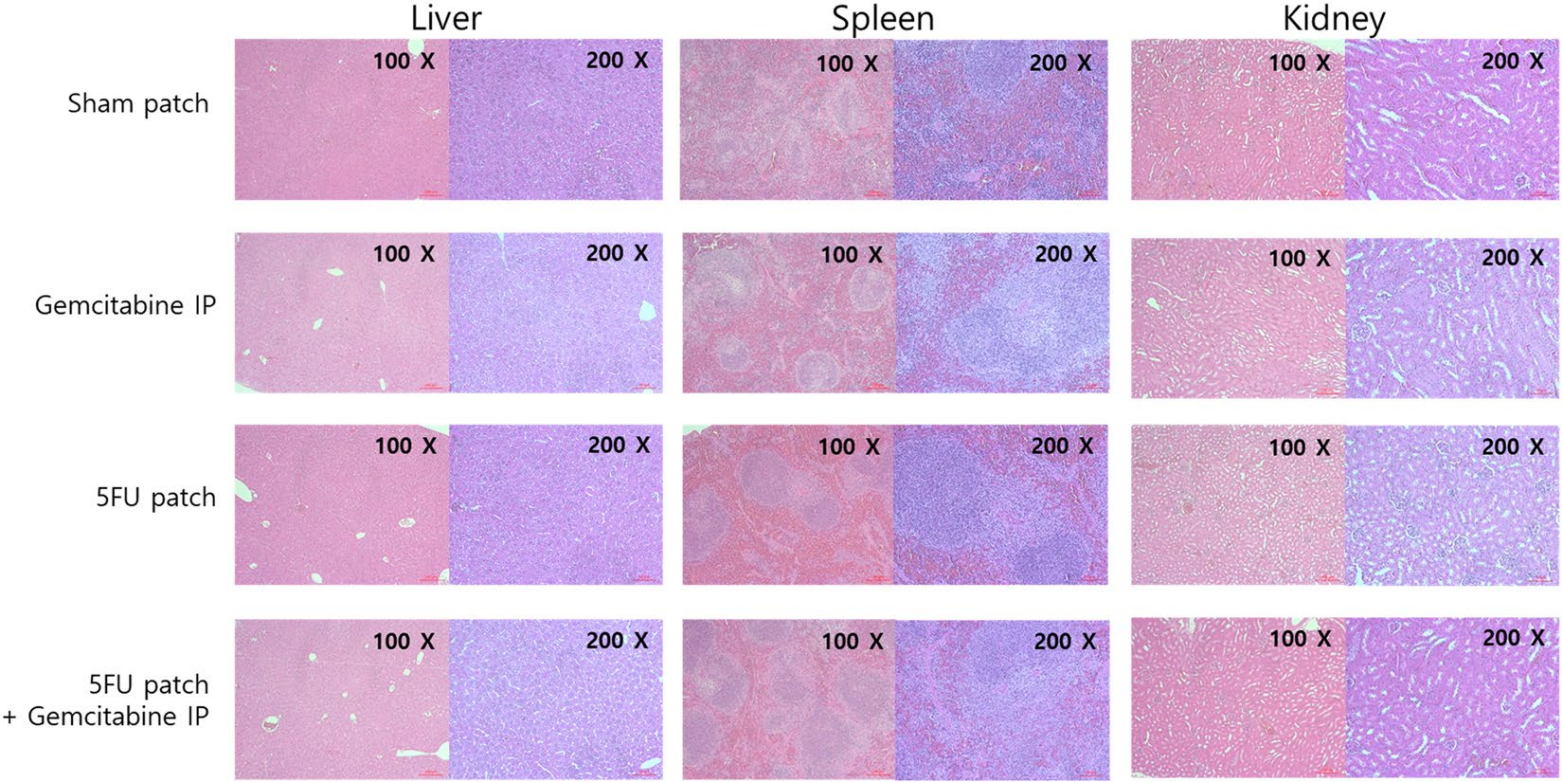
Histological evaluation of patch toxicity. Images of hematoxylin and eosin (H&E)-stained tissues of liver, spleen, and kidney from various treatment groups.

To evaluate the acute toxicity of 5-FU patch alone or in combination with gemcitabine, various parameters in the blood of the mice and histological changes in abdominal organs were analyzed. First, the blood of each group was analyzed on the third day after the start of treatment. Compared to the group treated with gemcitabine and 5-FU patch alone, the combination treatment showed a significant decrease in WBC, HCT, reticulocyte, and platelet in the blood. The degree of decreasing was similar among the mono-treatment groups, and WBC was less decreased in the 5 FU patch group. The analysis of liver and kidney function through mouse plasma showed no significant difference among the experimental groups. A further comparison of the blood on day 7 after treatment showed that the reduced hematological parameters were restored to normal level (Table 1). These results were also confirmed by H & E staining of liver, spleen, and kidney, which are associated with drug metabolism and immune response. There was no significant difference except that the lymphoid tissues of spleen were enlarged in the drug treatment groups (Fig. 6).

**Table 1:** Hematologic parameters from mice with various treatment groups.

	Unit	Sham patch	Gemcitabine IP	5-FU patch	5-FU patch + Gemcitabine IP	P value
**POD 3**						
WBC	10^3^/uL	4.85 ± 1.10	2.48 ± 0.25	2.95 ± 1.97	1.57 ± 0.52	*0.029*
NEU	%	37.23 ± 3.45	25.48 ± 7.71	30.97 ± 3.02	17.23 ± 5.51	*0.01*
LYMP	%	54.13 ± 6.16	69.18 ± 7.97	54.03 ± 14.07	68.33 ± 1.51	*0.079*
MONO	%	2.23 ± 1.45	1.68 ± 0.94	0.53 ± 0.35	0.43 ± 0.21	*0.088*
RBC	10^6^/uL	8.49 ± 0.05	8.93 ± 0.69	8.51 ± 0.33	8.07 ± 0.09	*0.139*
HGB	g/dL	12.77 ± 0.23	13.85 ± 1.01	13.00 ± 0.61	12.27 ± 0.12	*0.062*
HCT	%	40.37 ± 0.31	42.75 ± 1.63	39.97 ± 1.88	37.83 ± 0.45	*0.006*
Retic	%	4.29 ± 0.52	3.80 ± 0.40	1.32 ± 2.21	0.03 ± 0.02	*0.002*
PLT	10^3^/uL	1128.67 ± 122.03	823.50 ± 64.20	714.00 ± 177.05	493.00 ± 83.54	*0.001*
AST	IU/L	55.43 ± 7.73	58.83 ± 13.89	60.63 ± 9.93	69.73 ± 12.76	*0.506*
ALT	IU/L	26.80 ± 5.37	24.85 ± 7.52	20.40 ± 3.17	17.53 ± 2.27	*0.197*
TP	g/dL	3.75 ± 0.08	3.79 ± 0.36	3.88 ± 0.24	3.68 ± 0.12	*0.792*
BUN	mg/dL	20.03 ± 1.29	20.68 ± 2.04	20.30 ± 2.21	22.20 ± 4.47	*0.765*
Cr	mg/dL	0.29 ± 0.03	0.31 ± 0.08	0.26 ± 0.06	0.26 ± 0.04	*0.620*
**POD 7**						
WBC	10^3^/uL	4.75 ± 0.64	4.05 ± 0.80	2.00 ± 1.80	1.71 ± 0.65	*0.014*
NEU	%	42.03 ± 9.64	46.00 ± 11.59	24.00 ± 34.32	34.47 ± 23.53	*0.583*
LYMP	%	51.73 ± 9.65	45.13 ± 12.34	68.13 ± 37.68	44.90 ± 10.57	*0.475*
MONO	%	2.73 ± 0.65	3.28 ± 1.76	2.30 ± 3.81	3.40 ± 3.20	*0.944*
RBC	10^6^/uL	9.10 ± 0.31	8.72 ± 0.35	7.59 ± 0.25	6.71 ± 1.93	*0.044*
HGB	g/dL	14.00 ± 1.04	13.35 ± 0.58	11.57 ± 0.45	10.27 ± 3.00	*0.054*
HCT	%	45.27 ± 2.20	43.68 ± 2.00	37.83 ± 1.26	34.07 ± 9.77	*0.061*
Retic	%	4.46 ± 1.80	3.75 ± 0.87	1.95 ± 2.73	4.92 ± 1.02	*0.219*
PLT	10^3^/uL	854.00 ± 87.11	890.00 ± 69.15	1264.00 ± 405.28	1107.00 ± 330.45	*0.212*

Data are presented as average ± SD (n = 3). WBC: white blood cell, NEU: neutrophils, LYMP: lymphocytes, MONO: monocytes, RBC: red blood cell, HGB: hemoglobin, HCT: hematocrit, Retic: reticulocyte, PLT: platelet, AST: aspartate aminotransferase, ALT: alanine aminotransferase, TP: total protein, BUN: blood urea nitrogen, Cr: creatinine.

## Discussion

To cure and control cancer, a variety of studies have been performed to develop new therapies and delivery methods for established therapies^31–34^. Understanding the oncologic characteristics, such as chemotherapy resistance and the frequency and site of recurrence/metastasis is essential to develop effective strategies. In pancreatic cancer, which is characterised by low sensitivity to chemotherapy, a high local recurrence rate, and poor patient conditions after surgery, topical drug delivery could be a new alternative to overcome existing therapeutic limitations.

In this study, we constructed a PLLA-based drug patch using the electrospinning technique, which is a fiber production method in which sub-micron fibers form a matrix with interconnected pores^35^. The patent for electrospinning technology was registered in the United States in 1934, and there has been an increased interest in nanofiber technology in recent decades^30^. Studies on the protection and prolonged release of drugs are currently being conducted using electrospinning^36^. Natural polymers such as collagen, gelatin, fibrinogen, chitosan, and dextran, as well as biocompatible synthetic polymers such as PCL, PLA, PLGA, PVA and PEO, have been electrospun into scaffolds and used for tissue engineering^29^. The PLLA used in this study is a polyester of lactic acid or 2-hydroxypropionic acid, which is one of the most studied polymers in their class^37^. Because of its biocompatible and biodegradable nature, it is widely used in many fields of medicine including tissue engineering, resorbable sutures, fracture fixation, and drug delivery systems^38–42^.

In the present study, we first confirmed that the PLLA-based 5-FU patch could release the drug into the transplanted site by assessing its effect on inhibiting tumour growth *in vitro*. A patch without drug was used to confirm that the patch itself had no adverse side effects (sup Fig. 2). Application of the 5-FU patch showed that the drug was continuously released over 30 days, although most of the drug was released within the first 10 days. The pattern of early drug release observed suggests that the patch could be used for initial control of the tumour. In particular, considering that starting systemic chemotherapy at an appropriate time is difficult because of postoperative complications or poor conditions, intensive drug delivery through the topical patch could be of great help in preventing tumour recurrence^10–12^. However the degree of release of the drug could vary according to the scaffold type, the chemical nature of the drug, and the barriers associated with the route of administration. If drug release needs to be controlled according to the duration and purpose of the treatment, appropriate optimisation studies are needed^20^.

The drug patch prepared by electrospinning had a flexible nature, which was an advantage in our orthotopic tumour model in mice. Because tumours are produced in a variety of forms anywhere in the human body, the flexibility of the drug formulation and the ability to release drugs according to the conditions are important factors for drug patches. The drug loaded electrospun patch has the advantage of being freely adjustable according to the size or the site of the tumour, and it can therefore be used in various tumour models including pancreatic cancer.

The therapeutic effects of the drug patch were observed using IVIS and PET/MRI equipment. IVIS was used to confirm the bioluminescent signal of the BxPC3-luc tumours, and this equipment was especially helpful in cases where the tumour was inside the body. Biomolecular imaging systems are sensitive for the diagnosis of tumours and are useful for visualising the therapeutic effects of anticancer treatments^43–45^. PET/MRI can assess the internal state of the tumour and the degree of tumour activity in addition to its ability to identify tumours. The results of this experiment also showed the overall activity of the tumour, which may reflect tumour apoptosis, necrosis, and glucose metabolism. Although only one mouse was assessed by PET/MRI in this experiment, the results indicated the potential of the technique for assessing the therapeutic effect. Close monitoring of the tumours using various techniques is useful for establishing a treatment plan^46-48^

In an effort to maximize the therapeutic effect of drug patch, we have utilized a combination method with systemic chemotherapeutic drug. In particular, the method used in this study is characterized by the use of two drugs with difference in drug administration routes, therapeutic targets, and injection intervals. This combination method showed improved therapeutic effects compared with drug patch alone in the mouse xenograft model. These results suggest that local delivery could complement the effects of systemic anticancer drugs, and that simultaneous administration of two drugs with different mechanisms could improve patient outcome. Because tumour s have heterogeneous molecular and histological characters, it may be far more effective to employ diverse approaches than to treat tumour s with one drug^49^. The presentation of various therapies is considered to be effective not only in the primary treatment of tumours, but also in prevention of recurrence and metastasis of tumour and acquisition of resistance to anticancer drugs^50–52^. In order to maximize the effect of various therapies, it is necessary to design various characteristics of tumour, target of drug, working mechanism and pharmacokinetic factors. In addition, short-term toxicity was analyzed by hematological and histological analysis and long-term toxicity was analyzed by mouse weight. Combination group showed the highest toxicity due to the drug as the tumour treatment effect was the best. However, several hematologic toxicities identified immediately after treatment were found to recover over time. In addition, side effects in each single treatment group show interesting results. For the 5 FU patch and the gemcitabine IP group, the hematologic side effects on day 3 show similar results. Rather, WBC was found to be less reduced in the 5-FU patch group. However, considering the superior efficacy of local treatment using 5-FU patch, it has been confirmed that topical treatment to prevent recurrence of pancreatic cancer may be effective and it can be expected to play a role as a complement to existing systemic anticancer drugs.

In the present study, we examined the efficacy of local treatment of pancreatic cancer using an electrospinning drug patch and investigated the effects of combination treatment with a systemic anticancer drug. To maximise the efficacy of this combination therapy, it may be necessary to optimise the drug concentration and duration in further experiments. Through these studies, we hope to overcome the limitations of existing systemic anticancer drugs and accelerate the development of new therapeutic agents considering various patient conditions.

## Methods

### Study design

Using electrospinning technique, a drug patch containing 5-FU was prepared on poly-L-lactic acid (PLLA). Non-eluting drug patches were used as a control group, and the tumour killing effect between the two groups was compared *in vitro* as well as *in vivo* subcutaneous and orthotopic murine models. We compared the therapeutic effect and toxicities of different treatments among the systemic gemcitabine treatment group, 5- FU drug releasing patch group, systemic gemcitabine treatment plus 5-FU patch group in subcutaneous models. Gemcitabine, which is commonly applied in patients with pancreatic cancer, was used in a dose of 10 mg/kg via intra-peritoneal injection once a week. In gemcitabine alone group or combination group with a drug patch, gemcitabine was administered on the same day when the drug patch was implanted. Drug-releasing profile and cell cytotoxicity of the drug patch were confirmed. Tumour growth pattern and survival were monitored after subcutaneous and orthotopic tumour implantation by bio-molecular imaging. Drug toxicities were evaluated by assessing hematologic, hepatic, and renal functions. Histologic examination was performed as well. Detailed methods for each experiment are described separately.

### Construction and characterisation of electrospun drug patches

PLLA, methylene chloride, and 5-FU were purchased from Sigma-Aldrich (St. Louis, MO, USA). A blended solution was prepared by mixing PLLA and methylene chloride at 10% (w/v) concentration (weight ratio 2:1) at 37 °C. For the drug-eluting patchs, 5-FU (50 mg/mL) was added to the blended solution. The solution was loaded into a motorized pump syringe at a feeding rate of 2 mL/h and electrospun into a collector at 110 cm with a voltage of 15 kV. For the non-eluting patch (sham patch), the blended solution without 5-FU was electrospun under the same conditions. After electrospinning, the scaffolds were desiccated in a vacuum for 72 h. The fully dried patches were weighed uniformly at 20 mg, cut into squares, and stored at −20 °C^53^. A scanning electron microscope (SEM; Hitachi SU-6600, Hitachi, Tokyo, Japan) was used to analyse the morphology of the electrospun patches. The samples were platinum-coated by a sputter-coater and imaged using an accelerating voltage of 15 kV^53^.

### 5-FU release study

For the 5-FU drug release experiment, we first obtained a standard graph for 5-FU. To prepare various concentrations of the drug, 5-FU was serially diluted starting at 600 μM. A standard graph was obtained by measuring the absorbance at 265 nm using a UV–Vis spectrophotometer. To measure drug release from the electrospun patch, a 5-FU patch (5 mg, n = 3) was placed into 200 μL PBS at 37 °C, and PBS was exchanged every 3 days to collect the supernatant. UV absorbance at 265 nm of the supernatant collected over 30 days was measured, and the concentration of released drug was analysed using the standard graph^54,55^.

### Cell culture and therapeutic effect *in vitro*

BxPC3 (ATCC, Manassas, VA, USA), a human pancreatic cell line, was used to determine the response to 5-FU. The cells were cultured in RPMI-1640 medium (HyClone, Logan, UT, USA) supplemented with 10% v/v foetal bovine serum (Hyclone), 100 U/mL penicillin, and 100 μg/mL streptomycin (Gibco BRL, Grand Island, NY, USA) at 37 °C in 5% CO_2_ in a humidified incubator^55^. To confirm the response to 5-FU, a cell viability assay was performed. Cells were plated at a density of 3 × 10^3^ cells per well in a 96-well culture plate and incubated for 18 h before drug treatment (n = 3). The cells were then exposed to various concentrations of 5-FU (0.256 nM–100 μM) for 72 h. Relative cell viability was assessed using the cell counting kit-8 method (Sigma, St. Louis, MO, USA), and the median inhibitory concentration (IC_50_) was calculated. To assess the therapeutic effect of the 5-FU released from the drug patch, the supernatant was collected at an interval of 3 days, and 10 μL of supernatant was added to the cell medium in each well. Cell viability was determined at 72 h.

### Tumour xenograft model and monitoring of tumour size

The animal care and experimental protocols of this study were approved by the International Animal Care and Use Committee (IACUC) of the Laboratory of Animal Research at the Asan Medical Center, Seoul, Korea. (Permit Number: 2015-12-091). All experiments and methods were performed in accordance with relevant guidelines and regulations. The surgical procedures were performed in a specific-pathogen-free room. Six-week-old male BALB/c nude mice (n = 4 per group, Orient Bio Co., Seoul, Korea) were anaesthetised by intraperitoneal injection of 250 mg/kg 2,2,2-tribromoethanol (Sigma-Aldrich). For the subcutaneous model, the patch (20 mg, 8 mm × 8 mm) was inserted around the right flank region through the incision near the right leg and luciferase-transfected BxPC3 cells (2 × 10^6^ cells in matrigel) were subcutaneously implanted on the patches. Gemcitabine (50 mg/kg) was administered intraperitoneally once a week. Length (l, mm) and width (w, mm) were measured every 3 days using callipers, and tumour size was calculated as follows: Tumour size (mm^3^) = lw^2^/2. For the orthotopic model, luciferase-transfected BxPC3 cells (2 × 10^6^ cells in matrigel) were surgically implanted into the pancreas of mice (n = 5 per group). The abdomen was reopened after 7 days, and the drug patch was placed on the pancreatic tumour and marked by sutures. The mice were sacrificed on day 21, and the size of the tumours was calculated^56^. A tumour size of 300 mm^3^ was designated as an ethical end point, and the survival graph of the mouse was also confirmed based on this criteria^57^.

### IVIS *in vitro* and *in vivo*

To confirm the bioluminescence stability of luciferase-transfected Bxpc3 cell line, the signal intensity according to cell number was confirmed *in vitro*. 1 × 10^4^ to 1 × 10^6^ cells were seeded in a 24-well culture plate, and D-luciferin (150 μg/ml) was added to calibrate the IVIS (Caliper Life Science, PerkinElmer Inc., MA, USA.) For *in vivo* imaging, D-luciferin solution (150 mg/kg) was intraperitoneally injected 10 min before imaging. Bioluminescence imaging was performed using the IVIS^58^.

### PET/MRI in a subcutaneous model

PET/MRI fused imaging was performed using the nanoScan PET/MRI system (1T, Mediso, Hungary). Mice were fasted for 8 h before imaging, maintained at a constant body temperature, and injected intravenously via the tail vein with 6.5 ± 1.0 MBq in 0.2 mL of FDG. Mice were kept under anaesthesia (1.5% isoflurane in 100% O_2_ gas). The T1-weighted with Gradient-echo (GRE) 3D sequence (TR = 25 ms, TEeff = 3.4, FOV = 64 mm, matrix = 128 × 128) was acquired during the FDG uptake period. Static PET images were acquired for 10 min in a 1–5 coincident in a single field of view in the MRI range. Body temperature was maintained with a heating pad on the animal bed (Multicell, Mediso, Hungary) and a pressure sensitive pad was used for respiratory triggering. PET images were reconstructed by Tera-Tomo 3D in full detector mode with all the corrections on, high regularisation, and eight iterations. Three-dimensional volume of interest (VOI) analysis of the reconstructed images was performed using the InterView Fusion software package (Mediso, Hungary) and applying standard uptake value (SUV) analysis. The VOI was fixed in a sphere of 2 mm diameter, which was drawn for the tumour and muscle sites. The SUV of each VOI site was calculated using the following formula SUV mean = (tumour radioactivity in the tumour VOI with the unit of Bq/cc × body weight) divided by injected radioactivity^59^.

### Immunohistochemical staining

After sacrificing the mice, we removed the liver, spleen, kidneys, and tumours and fixed in 4% paraformaldehyde and embedded in paraffin. Paraffin blocks were cut into 4 μm sections and were reviewed histologically after hematoxylin and eosin (H&E) staining. For Ki-67 staining, after deparaffinization and antigenic retrieval, the slides were labeled with a monoclonal antibody against Ki-67 (cat M7240, clone MIB1, DAKO, Denmark). Labeling was detected using the avidin-biotin complex staining method. TUNEL staining was performed using *in situ* cell-death detection kit (Roche Diagnostics GmbH, Mannheim, Germany) according to the manufacturer’s protocol. The samples were mounted using Prolong Gold antifade mountant with DAPI^60^.

### Western blotting

Protein samples were extracted from frozen tumours removed from sacrificed mice. For whole-lysate extraction, RIPA Lysis and Extraction Buffer (Biosensing, Seongnam, Republic of Korea) supplemented with a protease-inhibitor cocktail was used. The protein concentration of each sample was determined using a BCA protein assay kit (Thermo Fisher Scientific). Afterwards, 10 μg of protein were separated on SDS PAGE, transferred to a nitrocellulose membrane, and probed with anti-caspase 3 (1:500; Abcam, USA) and anti-GAPDH antibody (1:1,000; Santa Cruz Biotechnology, CA, USA)^60^.

### Analysis of hematological parameters

Blood of mouse were taken from the abdominal vein. This experiment was performed in triplicate. Blood samples were collected for hematology determinations in tubes with ethylene diamine tetraacetic acid (EDTA) as an anticoagulant. Hematology determinations included white blood cell (WBC), differential leucocyte (neutrophil, lymphocyte, monocyte), red blood cell (RBC), hemoglobin (HGB), hematocrit (HCT), platelet (PLT), and reticulocyte using an Advia 120 Hematology analyzer (Bayer Healthcare, Myerstown, PA, USA). For chemistry analysis, 300 μl of blood were centrifuged for 15 mins at 3,000 rpm at 4 °C, and the plasma was carefully transferred to Eppendorf tubes. Aspartate aminotransferase (AST), alanine aminotransferase (ALT), total protein (TP), blood urea nitrogen (BUN), and creatinine (Cr) were analyzed.

### Statistical analysis

Statistical analyses were performed using SPSS for Windows, version 21.0 (IBM Corp Armonk, NY, USA). Comparisons between groups were performed using the T-test and ANOVA. P-values of <0.05 were considered statistically significant.

## Electronic supplementary material

supplementary Figure 1-5
