# Supplementary material for: Synergistic effect of a drug loaded electrospun patch and systemic chemotherapy in pancreatic cancer xenograft
Source: Sci Rep. 2017 Sep 28;7:12381. doi: 10.1038/s41598-017-12670-3 (PMC5620083; doi:10.1038/s41598-017-12670-3)

**1. Article type :** Original article

**2. Title**

Synergistic effect of a drug loaded electrospun patch and systemic chemotherapy in pancreatic cancer xenograft

**3. Names and initials of each author**

Eunsung Jun, MD, PhD^a,b^, Song Cheol Kim, MD, PhD^a^, Chan Mee Lee^a^, Juyun Oh^a^, Song Lee PhD^c^, In Kyong Shim PhD^c^

**4. Department and institution**

a Division of Hepato-Biliary and Pancreatic Surgery, Department of Surgery, University of Ulsan College of Medicine & Asan Medical Center, 388-1 Pungnap-2 Dong, Songpa-gu, Seoul, South Korea
b Department of Biomedical Sciences, University of Ulsan College of Medicine, 388-1 Pungnap-2 Dong, Songpa-gu, Seoul, South Korea
c Asan Institute for Life Science, University of Ulsan College of Medicine and Asan Medical Center, 388-1 Pungnap-2 Dong, Songpa-gu, Seoul, South Korea

**5. Address for correspondence**

Song-Cheol Kim, MD.PhD.

Department of Surgery, University of Ulsan College of Medicine & Asan Medical Center, 388-1 Pungnap-2 Dong, Songpa-gu, Seoul, South Korea.

Tel: +82-2-3010-3936 E-mail : drksc@amc.seoul.kr

**Supplementary Figure 1. Cell viability assay with 5-FU in BxPC3-luc cells.** To confirm the
response of BxPC3-luc cells to 5-FU, a viability assay was performed (IC_50_ = 1.15 µM). **Supplementary Figure 2. Cell viability assay with the sham patch in BxPC3-luc cells.** The viability of BxPC3-luc cells was confirmed using the sham patch, and there was no side effect related to growth inhibition and proliferation (** p > 0.05). **Supplementary Figure 3. Luciferase assay of BxPC3-luc cells.** (a). The IVIS was used to confirm the bioluminescent signals according to the cell number of BxPC3-luc. (b). The bioluminescent signal was quantified, and the correlation between cell numbers and bioluminescent signals was linear (R^2^ = 0.9869)

**Supplementary Figure 4. Comparison of tumour size in a subcutaneous tumour model.** Approximately 3 weeks after treatment with the patch, mice were sacrificed and tumour size was compared (n=4).

**Supplementary Figure 5. Western blotting of caspase 3 and GAPDH in a subcutaneous tumour model.** Protein levels of each group were compared by Western blotting.

**Supplement Figure 1**

**
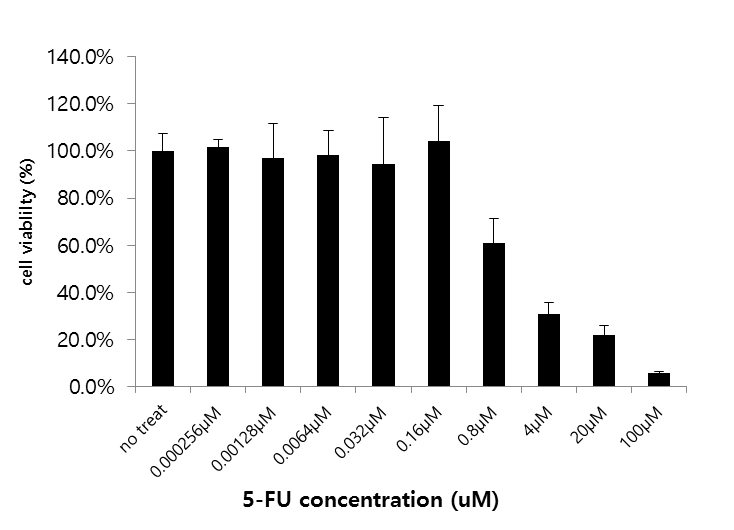
**

**Supplement Figure 2**

**
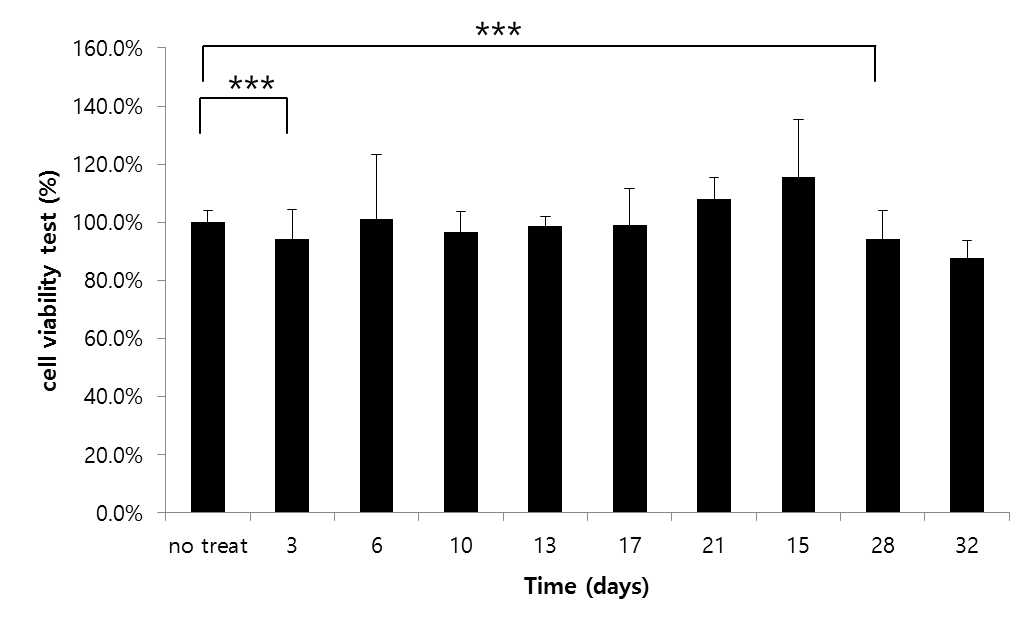
**

**Supplement Figure 3


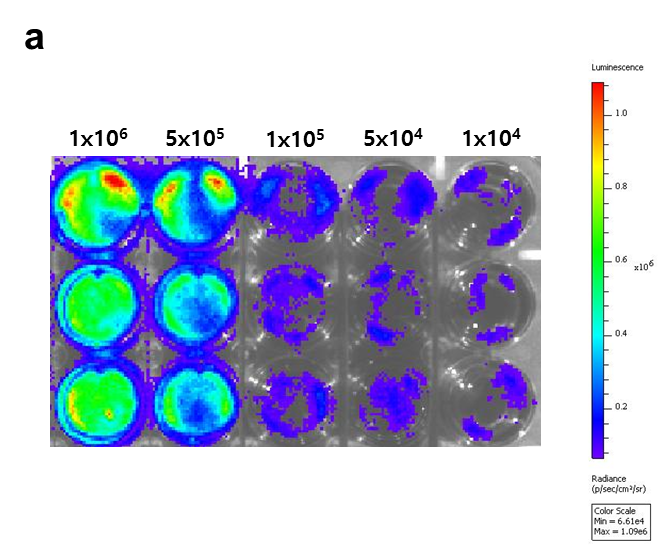
**

**
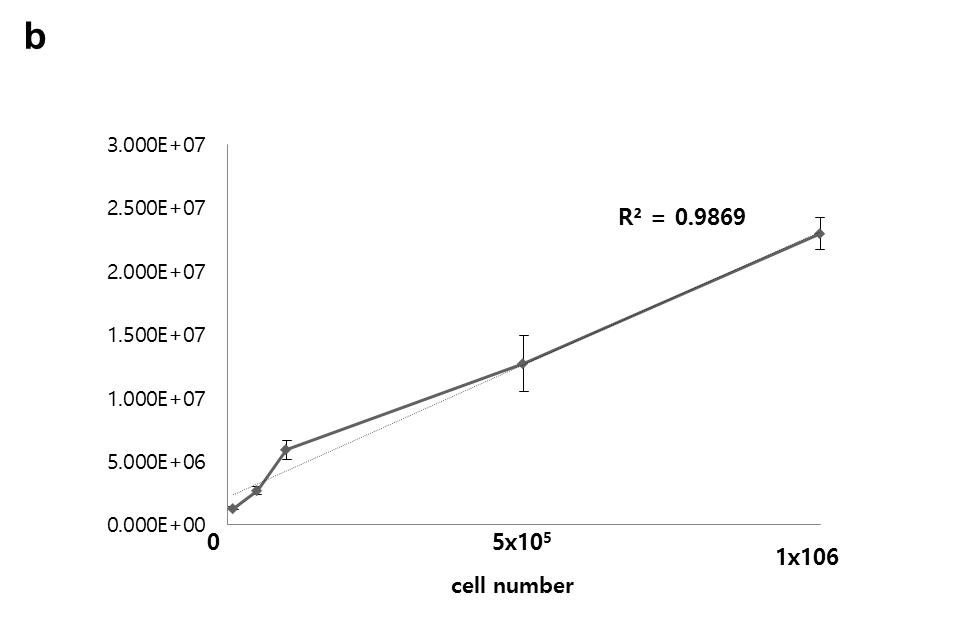
**

**Supplement Figure 4**

**
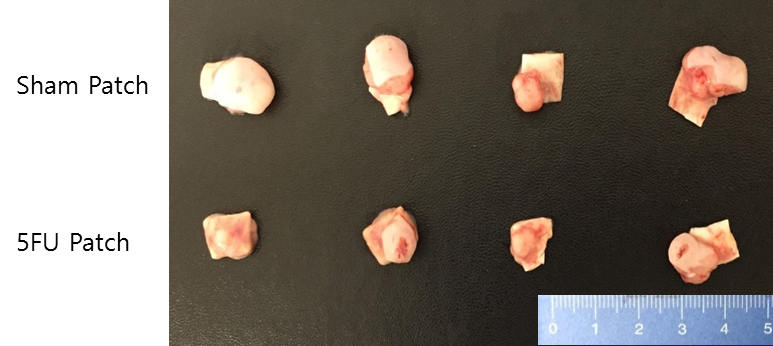
**

**Supplement Figure 5**


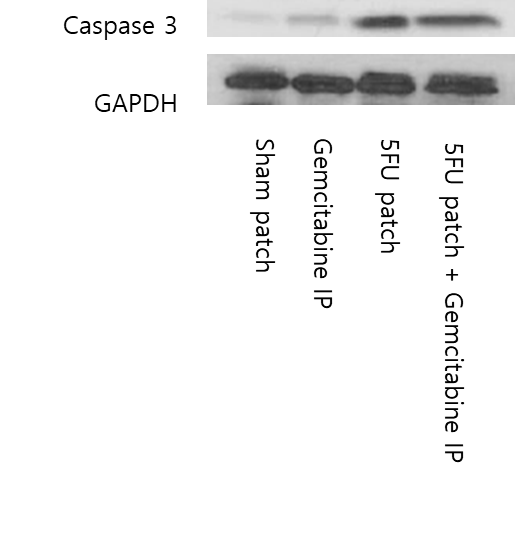

Supplement: Supplementary file 1 — supplementary Figure 1-5 [file 41598_2017_12670_MOESM1_ESM.doc]
